# New anatomical information on *Dsungaripterus weii* Young, 1964 with focus on the palatal region

**DOI:** 10.7717/peerj.8741

**Published:** 2020-04-01

**Authors:** He Chen, Shunxing Jiang, Alexander W.A. Kellner, Xin Cheng, Xinjun Zhang, Rui Qiu, Yang Li, Xiaolin Wang

**Affiliations:** 1 Key Laboratory of Vertebrate Evolution and Human Origins, Institute of Vertebrate Paleontology and Paleoanthropology, Chinese Academy of Sciences, Beijing, China; 2 CAS Center for Excellence in Life and Paleoenvironment, Beijing, China; 3 College of Earth and Planetary Sciences, University of Chinese Academy of Sciences, Beijing, China; 4 Laboratory of Systematics and Taphonomy of Fossil Vertebrates, Department of Geology and Paleontology, Museu Nacional/UFRJ, Rio de Janeiro, Brazil; 5 College of Earth Sciences, Jilin University, Changchun, China

**Keywords:** *Dsungaripterus*, Palatal region, Early Cretaceous, Pterosaurs

## Abstract

Pterosaur specimens with complete and well-preserved palatal region are rare. Here we describe new and previously collected specimens of the pterodactyloid pterosaur *Dsungaripterus weii* that are three-dimensionally preserved and provide new anatomical information for this species. Among the unique features is a lateral process of the pterygoid divided into two parts: an anterior thin, parabolic arc shaped element that separates the secondary subtemporal and the subtemporal fenestrae, followed by a dorsoventrally flattened portion that is directed inside the subtemporal fenestrae. The interpterygoid fenestrae join forming an irregular oval shape with two symmetrical posterior notches and a smooth anterior margin. Among all pterosaurs where the palate is known, the posterior configuration of the palate of *D. weii* is similar to some azhdarchoids, which is consistent with the suggested phylogenetic position of the Dsungaripteridae as closely related to the Azhdarchoidea. Furthermore, we identify symmetrical grooves on the lateral surface of the upper and lower jaws, that likely represent the impression of the edge of a keratinous sheath that would cover the upturned toothless rostrum during foraging activity, most likely consisting of hard elements, as has been previously assumed. Wear facets on the teeth also support this feeding mode.

## Introduction

*Dsungaripterus weii* is a member of the Dsungaripteridae and was first described in 1964 by Young (1964). After that publication, three complete skulls and some other elements were discovered, providing more anatomical information of this species (Young, 1973) and new skulls have been collected since 2006 by the IVPP. All of the fossil specimens were derived from the Early Cretaceous Tugulu Group of the Urho–Delunshan region near the northwestern margin of the Junggar Basin in the Xinjiang Uygur Autonomous Region of China (Young, 1964, 1973). Additional material attributed to *Dsungaripterus* was recovered from Wucaicheng, on the eastern margin of the Junggar Basin (Li & Ji, 2010). All materials of this pterosaur are three-dimensionally preserved and show a peculiar cranial and dental morphology that has been acknowledged by several researchers (Young, 1964, 1973; Kellner, 2003; Unwin, 2003; Lü et al., 2009; Li & Ji, 2010; Wu et al., 2017). Among the most striking features are the following: a well-developed sagittal crest that starts before the anterior margin of the nasoantorbital fenestra and extends above the occipital region; a robust anterior portion of the premaxilla, that tapers anteriorly and is upturned; a sub-rounded and comparatively small orbit that is positioned high up on the skull; bulbous teeth with broad and oval base; and lack of teeth on the tips of the skull and the mandible.

Recently, the palatal region of some pterosaur taxa has been described in more detail (Ösi et al., 2010; Pinheiro & Schultz, 2012; Kellner, 2013; Cheng et al., 2017), revealing some features that might show a phylogenetic signal. However, the specimens with well-preserved palatal region are quite rare, either flattened (Zhang et al., 2019) or too incomplete (Vullo et al., 2012). Despite the exceptional preservation, the palate of *Dsungaripterus* was never fully described.

Here we describe the palatal region of several specimens that have been originally collected by Young (1964) and new material collected in the Urho–Delunshan region since 2006, providing novel anatomical information particularly from the palatal region that complement the diagnosis of this pterosaur.

## Materials and Methods

The specimens collected in 1964 by Young and his colleagues that were published previously (Young, 1973) are the following: IVPP V 4063 (field number 64041-3), a nearly complete skull; IVPP V 4064 (field number 64045-2), an articulated skull and mandible; IVPP V 4065 (field number 64045-9), an incomplete skull, missing the anterior toothless tip that has a slightly crushed posterior region; IVPP V 26256 (field number 64045-5) and IVPP V 26561 (field number 64034-6), anterior part of a skull; IVPP V 26560 (field number 64041), a piece of the left side of a skull; IVPP V 26257 and IVPP V 26258, right and left pieces of the pterygoids and ectopterygoids. The new specimens recently collected by IVPP are the following: IVPP V 26259.1 and IVPP V 26259.2 are two pieces of the articulation of the right pterygoid and ectopterygoid from a broken skull. Those specimens are all from the Early Cretaceous Tugulu Group of the Urho–Delunshan region. Another specimen MCUGB 05-01-09 (Li & Ji, 2010) including articulated skull and mandible, which were laterally compressed and found on the eastern margin of the Junggar Basin, is analysed as well.

## Results

### Description

**Figure 1:**
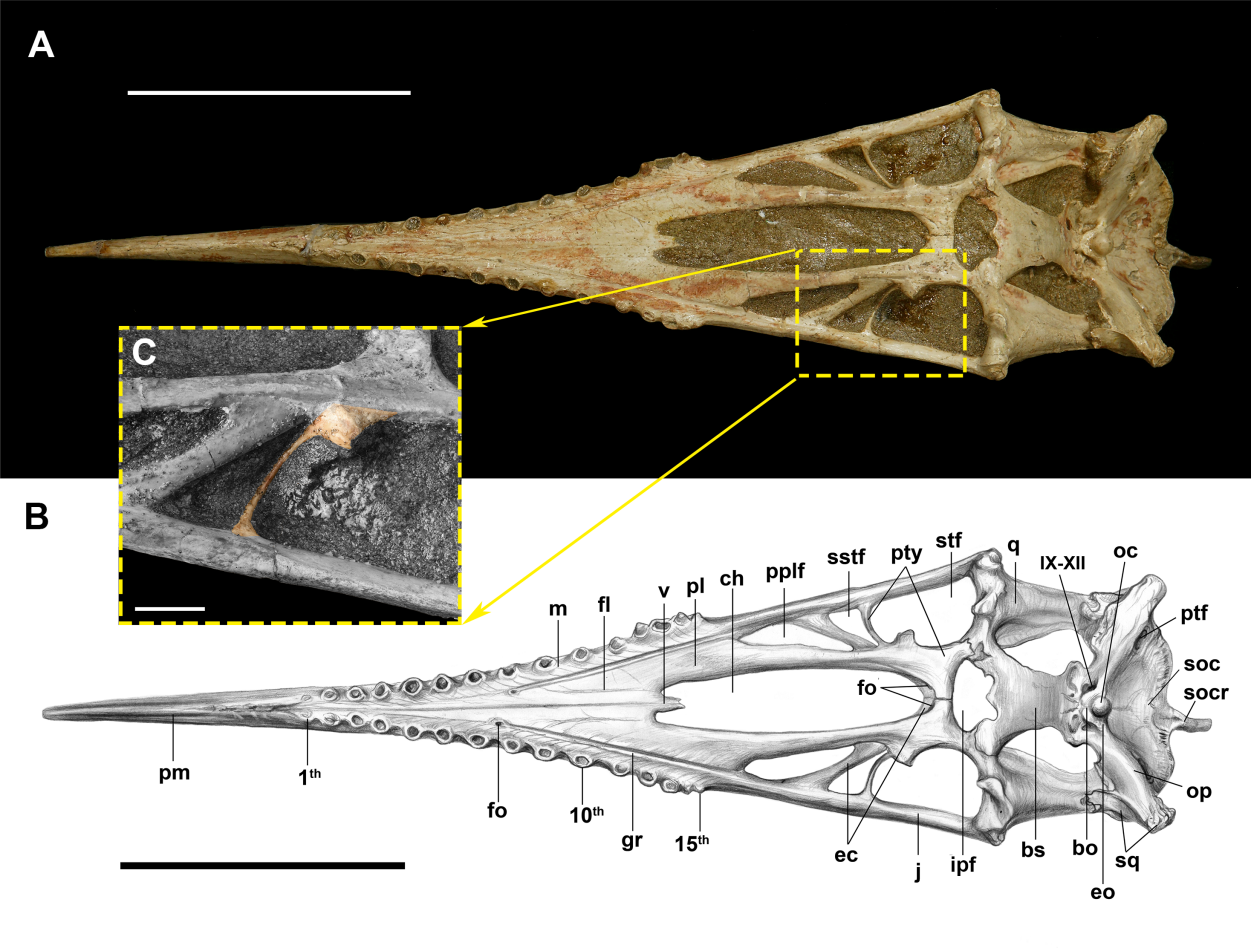
The palate of *Dsungaripterus weii* (IVPP V 4063) in ventral view. (A) Photo (Photo credit: Wei Gao) and (B) drawing (Sketch drawing credit: Xiaocong Guo), (C) close-up of the lateral process of the pterygoid (Photo credit: Wei Gao), showing in colour. Scale bars: 100 mm in (A) and (B), 10 mm in (C). Abbreviations: bo, basioccipital; bs, basisphenoid; ch, choana; ec, ectopterygoid; eo, exoccipital; fo, foramen; gr, groove; ipf, interpterygoid fenestra; j, jugal; m, maxilla; oc, occipital condyle; op, opisthotic; pl, palatine; pplf, postpalatine fenestra; ptf, posttemporal fenestra; pty, pterygoid; pm, premaxilla; pr, palatal ridge; q, quadrate; soc, supraoccipital; socr, supraoccipital crest; sq, squamosal; sstf, secondary subtemporal fenestra; stf, subtemporal fenestra; v, vomer; IX–XII, the ninth to twelfth cranial nerves.

**Figure 2:**
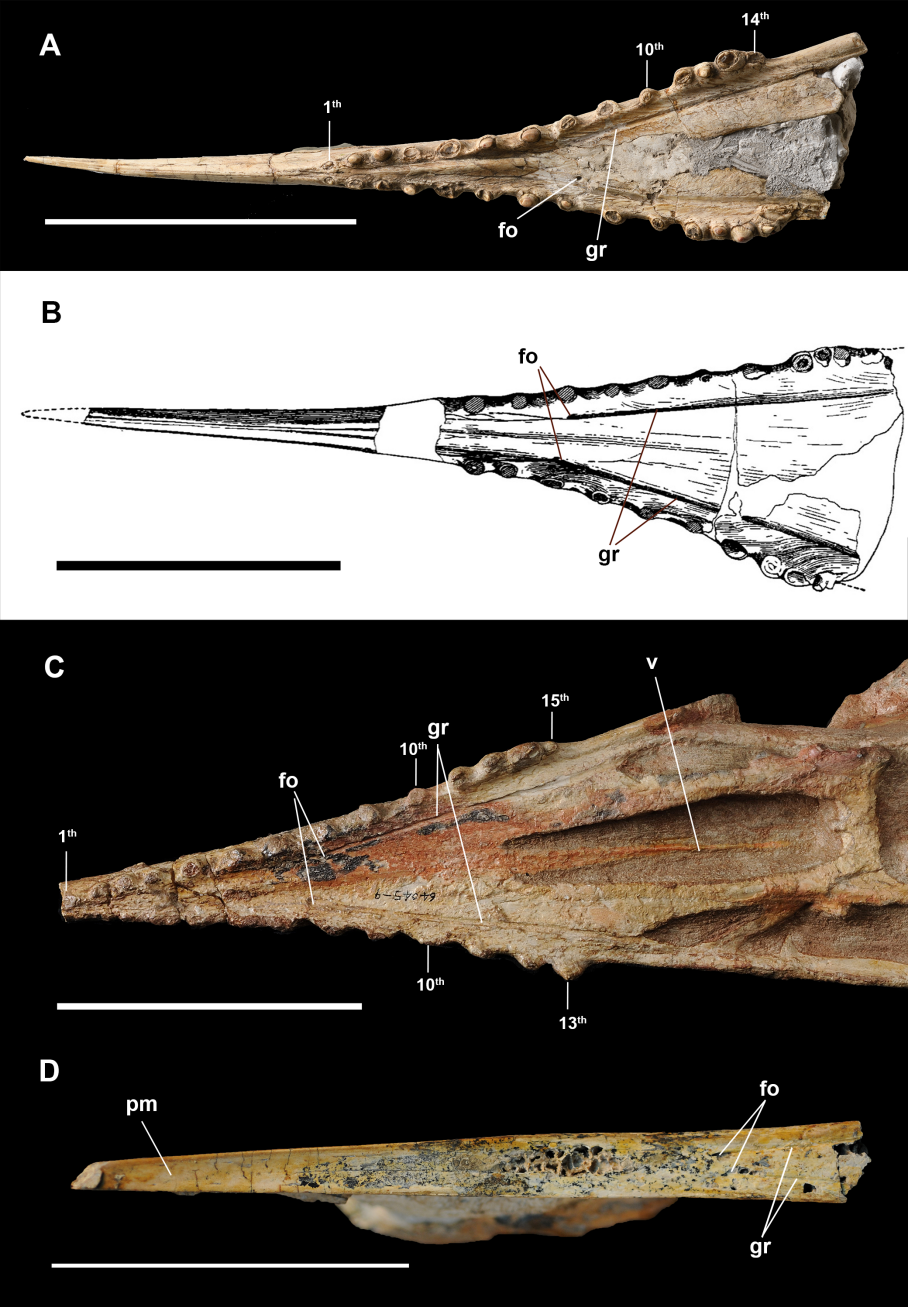
Anterior part of skulls in ventral view. (A) *Dsungaripterus weii* IVPP V 26256 (Photo credit: Wei Gao); (B) *Dsungaripterus weii*, drawing of IVPP V 2776, modified from Young (1964); (C) *Dsungaripterus weii*, IVPP V 4065 (Photo credit: Wei Gao); (D) *Caupedactylus ybaka*, MN 4726-V modified from Kellner (2013). Scale bars: 100 mm. Abbreviations: fo, foramen; gr, groove; pm, premaxilla; v, vomer.

From the *Dsungaripterus* specimens studied here, two of them show well preserved palatal region, with IVPP V 4063 being the most complete (Fig. 1). Young (1973) considered IVPP V 4064 as representing an adult and IVPP V 4063 as younger than the oldest one (IVPP V 4065). Moreover, due to the highly fused state of all cranial elements, IVPP V 4063 is here considered at least a subadult. The description of the palate is mainly based on the latter, with consideration on the anatomy of other specimens where appropriate (Fig. 2). We have also included some new observations on other portions of the skull and dentition.

#### Palatal openings

As observed in other pterosaurs, the largest opening on the palate is formed by the paired choanae, that are separated by the vomers. They are elongated (IVPP V 4063 length = 90 mm; IVPP V 4065 length = 118 mm), occupying about 28–32% of the length between the jaw articulation and the tip of the premaxilla, with rounded anterior and posterior margins.

The postpalatine fenestra is elongated, bordered anteriorly by the jugal process of the maxilla and the palatine and posteriorly by the ectopterygoid, giving it roughly a triangle shape appearance. It is followed by a secondary subtemporal fenestra (sensu Kellner, 2013), which is the smallest palatal opening, showing a roughly triangular shape, with a proportionally broad lateral margin and a very narrow medial margin (Fig. 1). The subtemporal fenestra is the second largest palatal opening, being roughly rectangular with slightly rounded anterior and posterior margins. The interpterygoid fenestrae are not separated from each other and have combined a roughly irregular oval shape with two symmetrical notches posteriorly.

#### Premaxilla and maxilla

**Figure 3:**
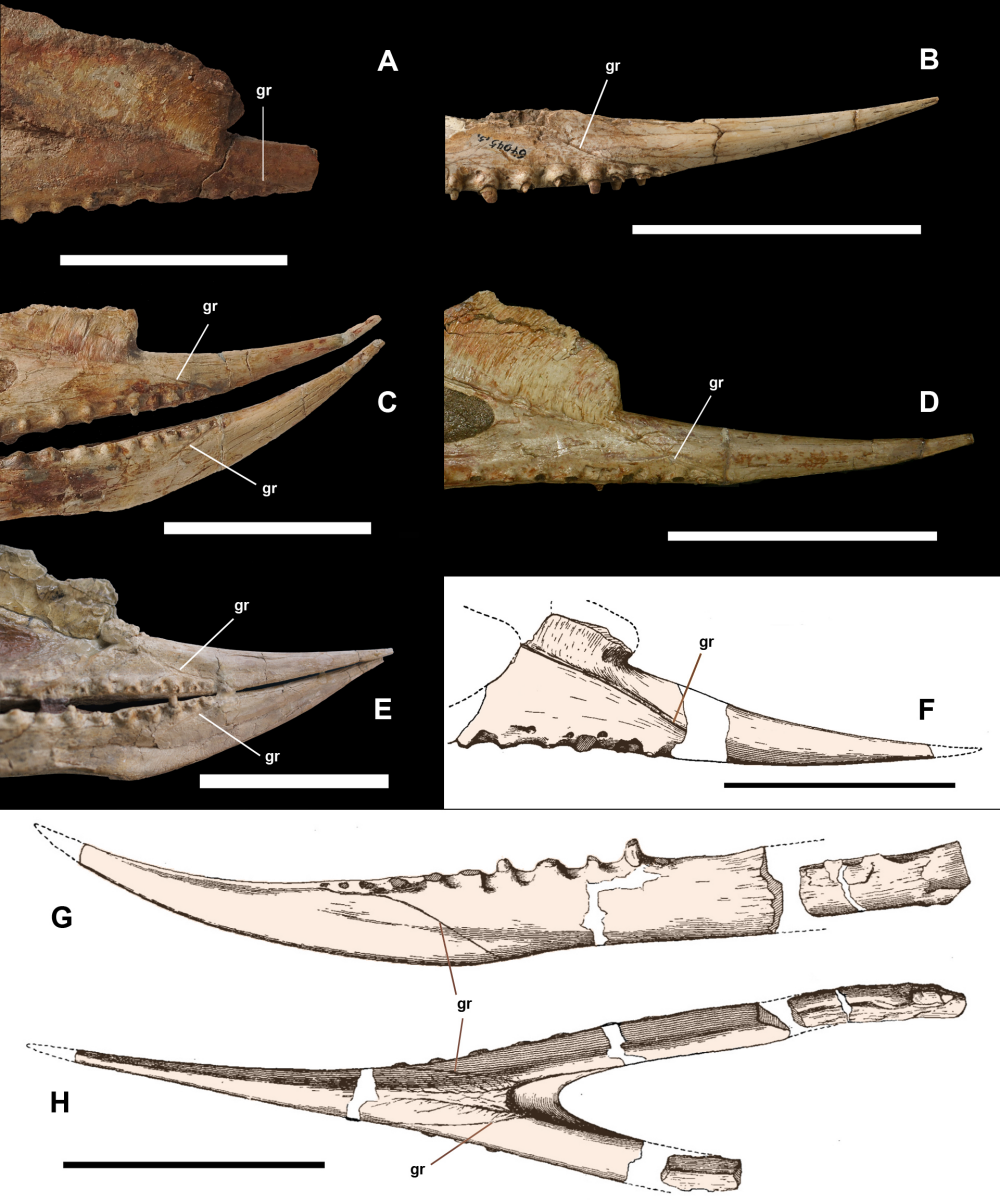
Anterior part of upper and lower jaws of *Dsungaripterus weii*. (A) The skull of IVPP V 4065 in right view (Photo credit: Wei Gao), (B) the skull of IVPP V 26256 in right view (Photo credit: Wei Gao), (C) the skull of IVPP V 4064 in right view (Photo credit: Wei Gao), (D) the skull of IVPP V 4063 in right view (Photo credit: Wei Gao), (E) the skull of MCUGB 05-01-09 in right view, (F) the skull of IVPP V 2776 in right view, modified from Young (1964), (G) the mandible of IVPP V 2776 in left view, modified from Young (1964), (H) the mandible of IVPP V 2776 in ventral view, modified from Young (1964). Scale bars: 100 mm. Abbreviations: fo, foramen; gr, groove.

**Figure 4:**
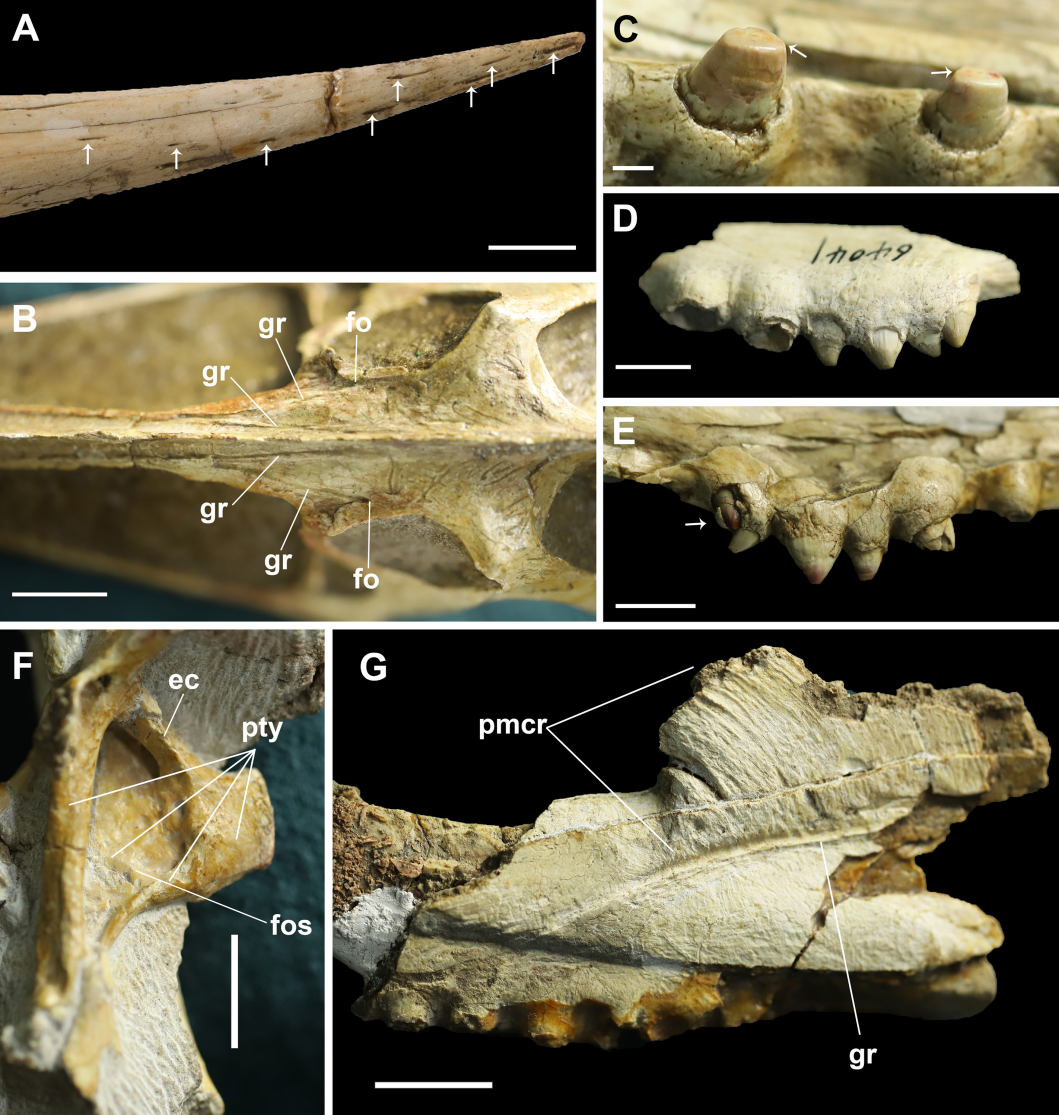
Details on the skull of *Dsungaripterus*. (A) Toothless part of rostrum. The arrows point to the foramina on the surface (Photo credit: Wei Gao). (B) Dorsal view of frontal region. (C) The teeth on the IVPP V 26256. The arrows point to the scratching surface on the teeth. (D) The left side of IVPP V 26560 has five teeth on the bony expansions. (E) The last tooth on the right side of IVPP V 26256 might be a replacement tooth, pointed by the arrow. (F) The dorsal portion of the pterygoid shows a developed fossa. This can be seen on the left side of IVPP V 26259.2. (G) IVPP V 26561, details of the groove and premaxillary sagittal crest. Scale bars: 10 mm in (A), (D), (E) and (F); 20 mm in (B) and (G); 2 mm in (C). Abbreviations: ec, ectopterygoid; fo, foramen; fos, fossa; gr, groove; pmcr, premaxilla crest; pty, pterygoid.

The strong, pointed rostrum of *Dsungaripterus* is formed by premaxillae and maxillae, two of which are fused in all specimens. In the description of the holotype (IVPP V 2776), Young (1964) noticed a groove on the lateral side of the skull that is directed from the base of the cranial crest to the first upper tooth and considered it being the suture between the premaxilla and the maxilla (Fig. 3F). Indeed, this groove runs along the base of the cranial crest, getting less pronounced and disappearing around the frontal (Figs. 4B and 4G), and the same groove is observed in all cranial material of this taxon known so far. The anterior part of the groove might very well be the indication of the premaxilla–maxilla limit (Young, 1964) and posterior part of this groove is consistent with the suture between the premaxillae and frontals, as seen in other pterosaurs (Wellnhofer, 1978; Campos & Kellner, 1985). A similar groove is also present in the lower jaw, mirroring the structure observed in the upper jaw (Figs. 3E and 3G). The groove on the lower jaw is not a suture, because it developed on the letteral side of the lower jaw, anterior to the end of the dentary symphysis. Therefore, it is possible that these grooves (in the upper and lower jaws) might also represent the impression on the bone of the edges of soft tissue that covered the rostrum and the crest rather than a suture. Only ontogenetically less mature individuals with unfused elements might give the clear limit between all the premaxillae and the maxillae.

The base of the cranial crest is positioned lower than the dorsal margin of the skull and it present the well-developed elongate sub-vertical striae and sulci, anteriorly curved (Fig. 4G). This kind of striae and sulci also present in the Hamipteridae, some Archaeopterodactyloidea and the Triassic pterosaur *Raeticodactylus*, and have been interpreted as a trait related to the attachment of the rhamphotheca (Holgado et al., 2019). There is another deep groove developed on the anterior area of the frontal, starting from a foramen (Fig. 4B) and running oblique to the sagittal plane that is here identified as the frontal–premaxillary suture.

Regarding the rostrum, at least 10 small foramina can be observed (Fig. 4A). There are also differences among the studied specimens, what might indicate individual variation or sexual dimorphism, the latter observed in at least one pterosaur species (Wang et al., 2014). The rostrum is complete (Figs. 3B–3E) and has almost the same length in four specimens (IVPP V 4063, IVPP V 4064, IVPP V 26256 and MCUGB 05-01-09). IVPP V 26256, however, shows a comparatively thinner anterior portion (Fig. 3B; Table 1) and might represent a female specimen, based on other studies (Wang et al., 2014).

**Table 1:** Measurements of the skulls of *Dsungaripterus weii* (in mm).

	IVPP V 4063	IVPP V 4064	IVPP V 4065	IVPP V 26256
Length of the skull (pr-sq)	390	461	(467) 367	/
Length of the palate	315^a^	390^a^	(380^a^) 295^a^	/
Length of the choana	90	/	118	/
Length of the tooth row	130^a^ 134.1^L^, 134.2^R^	150^a^ 157.7^L^, 159.4^R^	162^a^ 164.7^L^, 164.6^R^	147^a^ 142.7^L^, 144.0^R^
Length of the toothless part	(100) 95	95	/	100
Width of the rostrum at the first tooth	21.8	21.1	22.3	18.9
Depth of the rostrum at the first tooth	14.1	14,1	14.5	10.5
Number of the teeth on each side	15^L^, 15^R^	14^L^, 15^R^	15^L^, 13^R^	14^L^, 14^R^

**Notes:**

a From Young (1973).

L Left side.

R Right side.

/ Not preserve.

( ) Estimation.

On the ventral part, the portion of the palate anterior to the choanae was regarded as the vomer by Young (1973). However, this region in pterosaurs is generally composed by the maxillae and palatines (Wellnhofer, 1978, 1985; Campos & Kellner, 1985; Kellner, 1989; Bennett, 2001). There has recently been a discussion on the extension of the palatines in pterosaurs, with Ösi et al. (2010) arguing that what previous authors have regarded as the palatine are, actually, medial extensions of the maxillae (Pinheiro & Schultz, 2012), a interpretation has been challenged (Kellner, 2013). Although the fusion of the palatal elements prevents us to contribute to this discussion, we follow the general interpretation of the palatal region adopted by most authors (Wellnhofer, 1985; Campos & Kellner, 1985; Bennett, 2001) and consider most of the palate anterior before the choana to be formed by the maxillae and the palatines. In any case, it is unlikely that the vomer would form most of the palate of *Dsungaripterus* as Young (1973) assumed.

On the ventral side, a palatal ridge runs along the midline, starting from between the first and second alveoli, gets less pronounced towards the posterior region and disappears somewhere around the 9th alveoli (IVPP V 4063; IVPP V 4065, Figs. 1 and 2A). IVPP V 26256 shows a much more pronounced palatal ridge, which we regard as taphonomic.

The sutures of the maxilla and surrounding elements are obliterated. In IVPP V 4063 there are very faint lines that might have been the original sutures, but it is difficult to be certain (Fig. 1B). In any case, on each side there is one lateral deep groove that starts at a foramen positioned between the 7th and 8th tooth and runs posteriorly, parallel to the alveolar margin that might indicate the maxilla–palatine suture. At the posterior end, the maxilla has an elongated process (jugal process) that is overlain by the maxillary process of the jugal (suture not discernible) and forms the lateral margin of the postpalatinal fenestra.

#### Palatine

The palatines are fused with the surrounding palatal elements. Based on other pterodactyloid pterosaurs (Wellnhofer, 1985; Kellner, 2013; Howse & Milner, 1995), it is a long and narrow bone that contacts the maxilla anteriorly and laterally and posteriorly joins the pterygoid, participating in the anterior and lateral margins of the choanae.

#### Vomer

Only the anterior tip of the vomer is preserved in IVPP V 4063 (Fig. 1) and nearly complete in IVPP V 4065 (Fig. 2D). It starts as a dorsoventrally flattened element that tapers posteriorly, turning into a rod like structure. The anterior part fused with the palatine. Young (1973) mentioned that the parasphenoid might be anteriorly connected with the posterior part of the vomer, but there is no evidence of this in the available specimens.

#### Pterygoid

**Figure 5:**
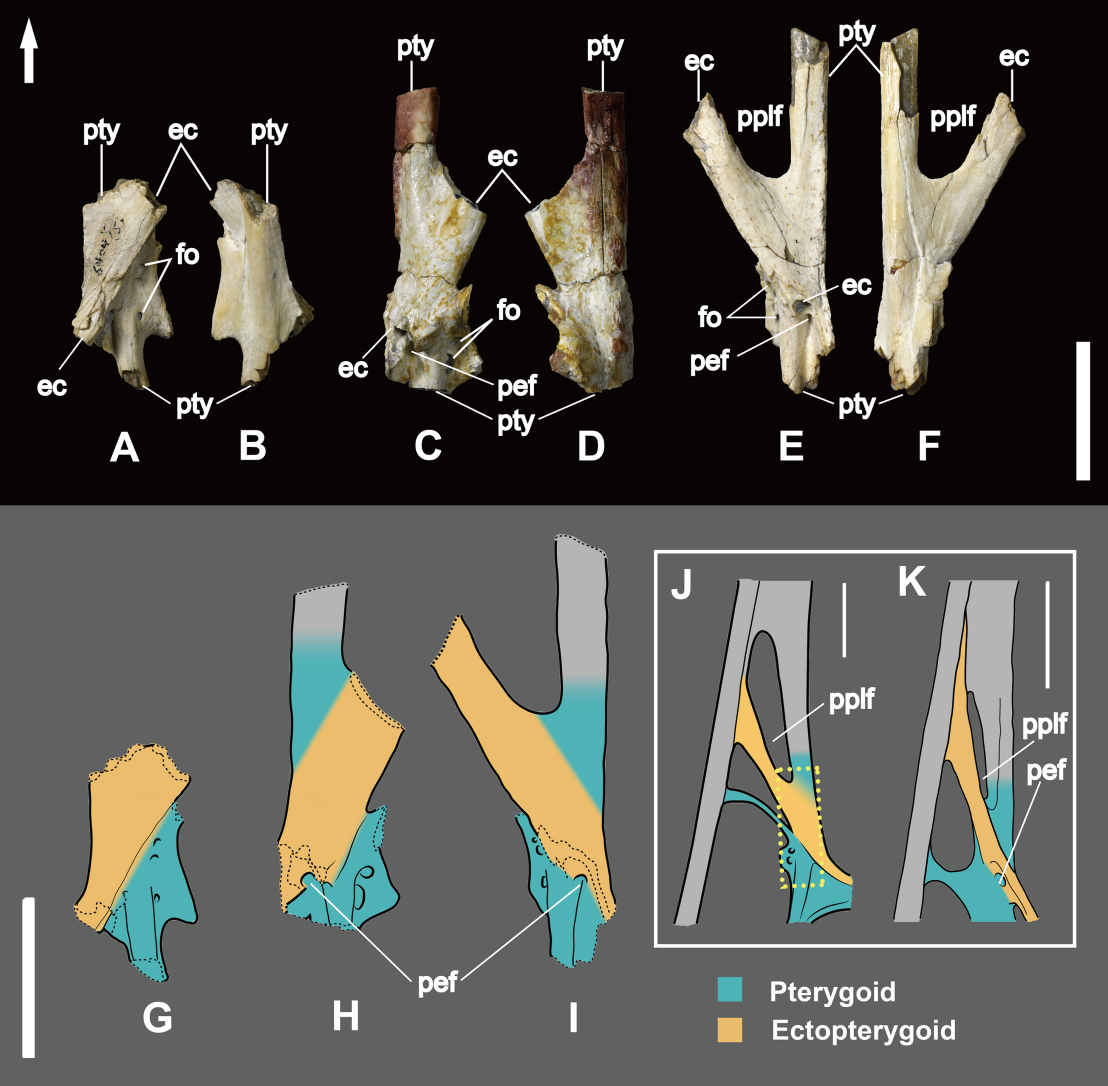
Part of the pterygoid and ectopterygoid. (A), (B) and (G): right side of *Dsungaripterus weii* IVPP V 26257 (Photo credit: Wei Gao); (C), (D) and (H): right side of *Dsungaripterus weii* IVPP V 26259.1 (Photo credit: Wei Gao); (E), (F) and (I): left side of *Dsungaripterus weii* IVPP V 26258 (Photo credit: Wei Gao); (J) line drawing of partial palate of *Dsungaripterus weii* on left side, the line of dashes in yellow shows the place where the IVPP V 26258 supposed be; (K) left side of *Caupedactylus ybaka* MN 4726-V, modified from Kellner (2013). (A), (C), (E), (G), (H), (I), (J) and (K) in dorsal view; (B), (D) and (F) in ventral view. Scale bars: 20 mm. The arrow points anteriorly. Abbreviations: ec, ectopterygoid; pplf, postpalatine fenestra; fo, foramen; pef, pterygoid-ectopterygoid foramen; pty, pterygoid.

Based on other pterosaurs (Wellnhofer, 1991a; Kellner, 2013), the pterygoid is an irregularly shaped bone which is fused with the surrounding elements in all available specimens, including the opposite pterygoid (Fig. 1). It shows three processes, with the anterior forming the lateral margin of the choana. Posterior to the anterior process, the main pterygoid body developed a lateral process that is divided into two parts, a feature so far unique to *Dsungaripterus weii* (Fig. 1). The most anterior of these two parts is a thin, parabolic arc shaped element with expanded ends that separates the secondary subtemporal and the subtemporal fenestrae (Figs. 1 and 2D). The second part of this process is dorsoventrally flattened and directed inside the subtemporal fenestrae. IVPP V 26257, IVPP V 26259.1 and IVPP V 26258 show the dorsal view of the part of the lateral process that fused with main pterygoid body, revealing the presence of 2–3 foramina (Fig. 5). They do not appear to be pneumatic as are commonly observed in other parts of the pterosaur skeleton (Buchmann & Rodrigues, 2019).

**Figure 6:**
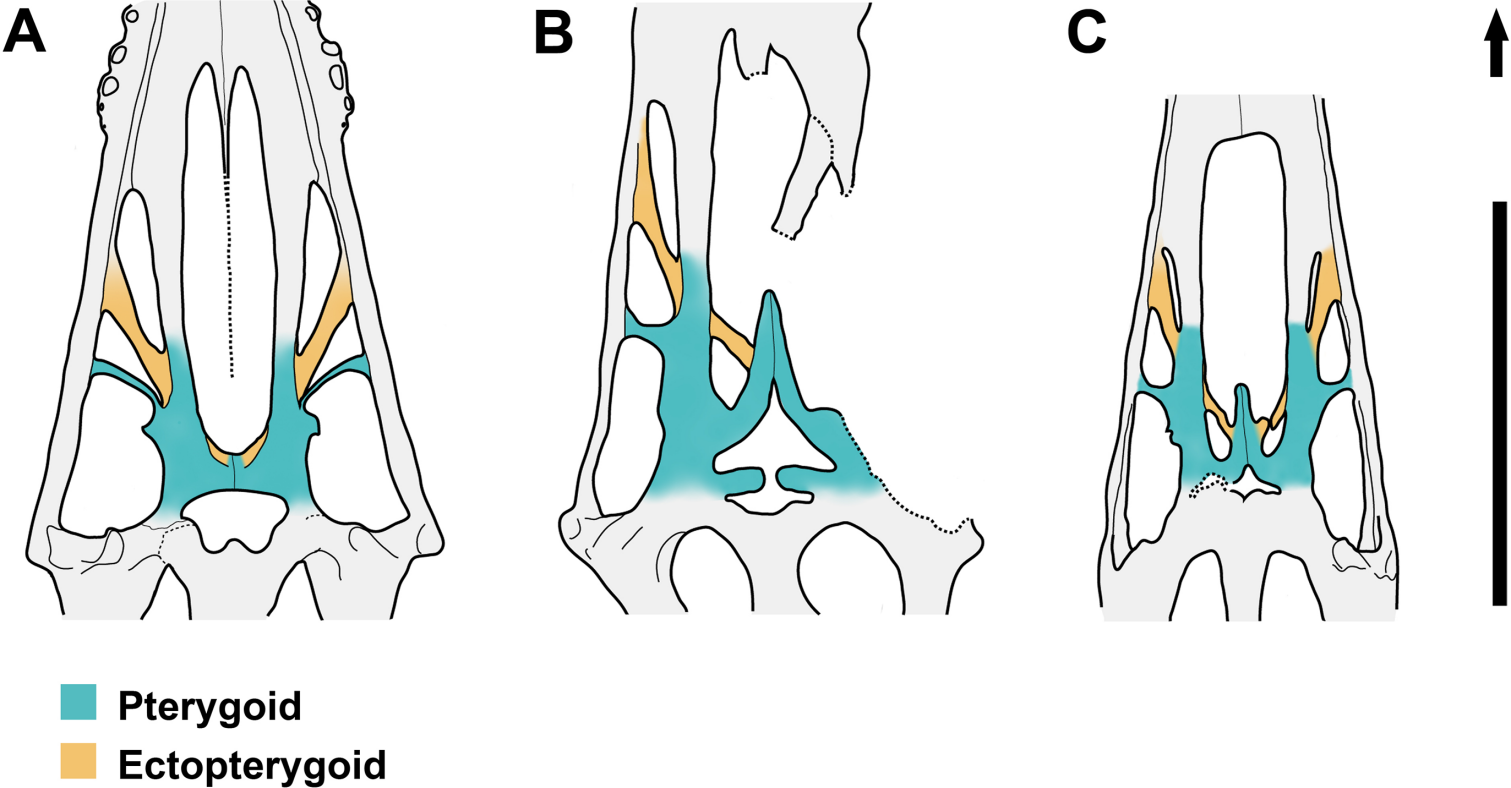
Line drawings of the posterior part of the palate in ventral view. (A) *Dsungaripterus weii* IVPP V 4063; (B) *Tupuxuara leonardii* IMCF 1052, modified from Pinheiro & Schultz (2012); (C) *Caupedactylus ybaka* MN 4726-V, modified from Kellner (2013). Scale bar: 20 mm. The arrow points anteriorly.

Posterior to the lateral process, the pterygoid forms a dorsoventrally flattened medial process that joins medially the opposite pterygoid, forming the anterior margin of the interpterygoid fenestra (Fig. 1). The posterior part of the pterygoid is fused with the quadrate and the basisphenoid (Figs. 1 and 6A). In IVPP V 26259.2 the dorsal portion of the left pterygoid is exposed a developed fossa (Fig. 4F).

#### Ectopterygoid

The ectopterygoid is preserved in several specimens, and can be observed from the dorsal view in IVPP V 26257, IVPP V 26259.1, IVPP V 26259.2 and IVPP V 26258 (Figs. 4F and 5). It is a flattened element that forms the posterolateral margin of the postpalatine fenestra and the anteromedial margin of the secondary subtemporal fenestra (Fig. 1). Laterally this bone is fused with the jugal. Medially it overlies the dorsal part of the pterygoid and reaches the posterior margin of the choana. A foramen is present on the contact area with the pterygoid.

The ectopterygoid bar that separates the postpalatine from the secondary subtemporal fenestra is hollow, with a ~1 mm thick cortex. Laterally this bone is fused with the jugal and medially it overlays and fuses with the pterygoid (Figs. 1 and 5A–5I).

A foramen (here named the pterygoid-ectopterygoid foramen) is open on the lateral side of posterior part of the ectopterygoid and faces towards the lateral-posterior ventral side and it can be seen from the intersectional fragments in dorsal view (Figs. 5C, 5E, 5H and 5I). This foramen connects to the cavity of the ectopterygoid and it is not visible in ventral view.

#### Dentition

The posterior expansion of the maxilla is a diagnostic feature of *Dsungaripterus* (Kellner, 2003, 2004; Unwin, 2003; Andres & Myers, 2013) and is present in all specimens except for the right side of IVPP V 4065 where no perceptible bony expansion can be identified (Fig. 2D). This expanded portion of the maxillae bears four or five teeth (Fig. 4D), with the two first larger than the previous maxillary teeth, which are followed by two or three smaller teeth. The number of teeth on each side of the skull varies between 14 and 15, with the right side of IVPP V 4065 having 13 teeth (Figs. 1 and 2). The teeth in the IVPP V 26256 have a clear smooth wear surface, parallel to the palatal plate (Fig. 4C). The alveoli show a thick rim and are raised from the alveolar margin (Martill et al., 2000; Hone, Jiang & Xu, 2017). There is a marked depression on the lateral side between each alveolus that receives the respective lower tooth. Judging from the diameter of the alveoli, the teeth increase in size posteriorly until the 5th tooth position, decreasing thereafter until the bony expansions. Additionally, a possible replacement tooth is preserved on the right side of IVPP V 26256 (Fig. 4E).

## Discussion

The available material of other dsungaripterids species such as *Noripterus complicidens* (Young, 1973; Lü et al., 2009; Hone, Jiang & Xu, 2017), ‘*Phobetor’* (Bakhurina, 1982; Wellnhofer, 1991b; Bakhurina & Unwin, 1995) and *Lonchognathosaurus acutirostris* (Maisch, Matzke & Sun, 2004) provide little information on the palate region. The material of *Domeykodactylus ceciliae*, another purported dsungaripterid, lacks the palatal region entirely (Martill et al., 2000).

*Noripterus* (some material also argued as ‘*Phobetor’* (Hone, Jiang & Xu, 2017)) has no groove or ridge on the anterior portion of the palatal surface (Lü et al., 2009), which is present in *Dsungaripterus*. *Lonchognathosaurus*, which is based on a laterally compressed anterior part of a skull, differs by lacking any foramen or groove on the palate (Maisch, Matzke & Sun, 2004). No detailed information of the palate can be retrieved from the published illustration of ‘*Phobetor’* Wellnhofer (1991b).

The palate of *Dsungaripterus* is different from that of ctenochasmatids including *Aurorazhdarcho micronyx* (BSP 1936 I 50, Wellnhofer, 1970; Bennett, 2013), *Gnathosaurus subulatus* (BSP 1951.84, Wellnhofer, 1970), *Plataleorhynchus streptophorodon* (BMNH R.11975, Howse & Milner, 1995), *Liaodactylus primus* (PMOL AP0031, Zhou et al., 2017). The postpalatine fenestra in all ctenochasmatids where the palate is more rounded, with the medial process of pterygoid points anteriorly. Both *Gnathosaurus* and *Aurorazhdarcho* have a pointed anterior margin of the choanae, the lateral process of pterygoid dose not separate the subtemporal fenestra. The *Gnathosaurus* has a relatively smaller choanae, occupying about 22% of the length of palate, differing from 28% to 32% observed in *Dsungaripterus*. *Plataleorhynchus* only preserved rostral part of the skull and the rostrum bears a terminal spatula, which is totally different from *Dsungaripterus*. *Liaodactylus* developed the thin lateral process of the pterygoid dividing the subtemporal fenestra, make the rounded medial margin of the secondary subtemporal fenestra, not as in *Dsungaripterus*.

Although *Hamipterus tianshanensis* also shows a developed lateral process of the pterygoid separating the subtemporal fenestra (Wang et al., 2014), the extremely thin and slender shape differs from *Dsungaripterus*. The postpalatine fenestra is relatively smaller (occupying about 4% of the length of palate) and more rounded than *Dsungaripterus* (about 12–13%).

All of the specimens attributed to the Anhangueridae where the palate is known, such as *Tropeognathus mesembrinus* (Wellnhofer, 1987), *Anhanguera* (Campos & Kellner, 1985; Wellnhofer, 1991a; Pinheiro & Schultz, 2012; Pinheiro & Rodrigues, 2017) have a more rounded and relatively smaller postpalatine fenestra than *Dsungaripterus*. They also show pointed anterior margin of interpterygoid fenestra differing from *Dsungaripterus*. Although the specimen of the anhanguerid *Maaradactylus kellneri* (Bantim et al., 2014) only shows the palatal region anterior to the postpalatine fenestra, the anterior part of it expands laterally in a spoon shape, not as in *Dsungaripterus*. Furthermore, the end of the lateral process, which join the pterygoid, is more robust and knob-like compared to other taxa such as *Anhanguera* (Wellnhofer, 1991a; Pinheiro & Rodrigues, 2017).

The palate of *Dsungaripterus* differs from the one of the istiodactylid *Hongshanopterus lacustris* (Wang et al., 2008), the pteranodontid *Pteranodon* (Eaton, 1910; Bennett, 2001) and the nyctosaurid *Nyctosaurus gracilis* (Williston, 1902). Among the differences, all cited taxa lack the developed lateral process of pterygoid that divides the subtemporal fenestra and the postpalatine fenestrae start posterior to half the length of the choanae. Furthermore, *Pteranodon* and *Nyctosaurus* have significant smaller postpalatine fenestrae and apparently lack an ossified vomer separating the choana, and *Pteranodon* shows a very small interpterygoid opening, the smallest within Pterosauria so far. The end of the lateral process of the pterygoid is more robust and knob-like in *Dsungaripterus* than in *Hongshanopterus* (Wang et al., 2008).

The posterior part of the palate of *Dsungaripterus* has more similarities with the azhdarchoids *Caupedactylus ybaka* (Kellner, 2013) and *Tupuxuara leonardii* IMCF 1052 (Witton, 2009; Pinheiro & Schultz, 2012) (Fig. 6). All show the ectopterygoid overlaying the pterygoid dorsally and fused with the medial pterygoid process posteriorly. They also show a developed lateral process of the pterygoid dividing the subtemporal opening in two fenestrae, with the anterior part called secondary subtemporal fenestra (Kellner, 2013). These shared palatal features between *Dsungaripterus* and azhdarchoids agrees with their close relationships advocated by several phylogenetic analyses (Kellner, 2003, 2004; Unwin, 2003; Wang et al., 2009; Lü et al., 2010; Pêgas, Leal & Kellner, 2016; Holgado et al., 2019; Kellner et al., 2019). Notwithstanding, there are differences among the palate of these three species. *Caupedactylus* has a unique slit-like and narrow postpalatine fenestra and in *Tupuxuara* this opening is more rounded, not triangular as in *Dsungaripterus*. In *Caupedactylus*, the ectopterygoid reaches the anterior edge of postpalatine fenestra (Fig. 5K) while in *Dsungaripterus*, it lies posterior to the edge of this palatal opening (Fig. 6A). Furthermore, *Dsungaripterus* and *Caupedactylus* developed a foramen on the ectopterygoid and a pair of grooves and foramina on the anterior portion of the palate. Additionally, the system of branching grooves that radiate from the foramina positioned in the anterior portion of the palatal ridge in *Tupuxuara leonardii* (Kellner & Campos, 1994) are different from the grooves and the foramen in *Dsungaripterus* and *Caupedactylus*. A small foramen is present at the contact area between the medial pterygoid process and the posterior part of the ectopterygoid in *Dsungaripterus*, while in *Tupuxuara* and *Caupedactylus* there is a fenestra. The interpterygoid fenestra of *Dsungaripterus* differ from the ‘heart-shape’ outline of *Caupedactylus ybaka* (Kellner, 2013) and *Tupuxuara leonardii* (Witton, 2009; Pinheiro & Schultz, 2012), by being irregular with two symmetrical posterior notches.

Another interesting observation are the lateral grooves on the upper and lower jaw that mirror each other. Despite the fact that the one of the upper jaws is in the position where the suture between premaxilla and maxilla is expected, it might have been the insertion point of a horny covering (Fig. 3). Young (1964) suggested that the rostrum of this pterosaur might have been covered by a horny sheath, a possibility that is consistent with the presence of the grooves.

Regarding the function of the rostrum, based on the peculiar dentition formed by bulbous, small and strong teeth, combined with the narrow and edentulous jaw tips, several researchers advocated that *Dsungaripterus* probably grabbed, probed or dislodged shellfish and hard-shelled insects by using their upturned pincerlike jaws in shallow water or on mudflats, like probing birds do nowadays (Young, 1964; Wellnhofer, 1991b; Unwin, 2005; Witton, 2013). Furthermore, the expanded opisthotic processes in *Dsungaripterus* might have anchored strong neck muscles, consistent with the hypothesis of shellfish-eating and extracting or dislodging prey (Habib & Godfrey, 2010; Witton, 2013). Our interpretation supports that *Dsungaripterus* might have had the rostrum covered by a thick horny sheath (Young, 1964; Wellnhofer, 1991b; Unwin, 2005; Habib & Godfrey, 2010; Witton, 2013), that protected the anterior toothless portion of the rostrum from abrasion during preying, which is consistent with the previous feeding hypothesis proposed by other authors.

Lastly, the presence of at least two pterosaur taxa in the same localities (*Dsungaripterus weii* and *Noripterus complicidens*) might be a case of sympatry of pterosaur species, as has been recently reported in Brazil (Kellner et al., 2019). More field work in necessary regarding the Chinese deposits to confirm if these two taxa indeed occur in the same horizon or if they come from different layers.

## Conclusions

*Dsungaripterus weii* has a unique lateral pterygoid process that is divided into two parts. The most anterior is a thin, parabolic arc shaped element that separates the secondary subtemporal and the subtemporal fenestrae. The second part of this process is dorsoventrally flattened with some foramina on dorsal side and directed inside the subtemporal fenestrae. The interpterygoid fenestrae have jointly an irregular oval shape with two symmetrical posterior notches, not reported in any pterodactyloid before. The number of teeth on each side of the upper jaw varies between 14 and 15 in most cases, with the last four or five positioned on an expanded area of the maxilla, unique to this species. The groove lines on the lateral side of the upper jaw are mirrored on the lower jaw and might be the impression of the posterior edge of a horny sheath that covered the toothless anterior portion of the rostrum. The posterior configuration of the palate of *Dsungaripterus weii* is similar to the azhdarchoids *Caupedactylus ybaka* and *Tupuxuara leonardii*, what is consistent with its suggested phylogenetic position as closely related to the Azhdarchoidea.

## Institutional Abbreviations

BMNH: British Museum Natural History, London, UK
BSP: Bayerische Staatssammlung für Paläontologie und historische Geologie, Munich, Germany
IMCF: Iwaki Coal and Fossil Museum, Iwaki, Japan
IVPP: Institute of Vertebrate Palaeontology and Palaeoantropology, Beijing, China
MCUGB: Museum of China University of Geosciences (Beijing), China
MN: Museu Nacional, Universi-dade Federal do Rio de Janeiro, Rio, Brazil
PMOL: Palaeontological Museum of Liaoning, Liaoning, China

## Additional Information and Declarations

### Data Availability

The following information was supplied regarding data availability: The specimens are stored in Institute of Vertebrate Palaeontology and Palaeoantropology, Beijing, China: IVPP V 4063, IVPP V 4064, IVPP V 4065, IVPP V 26256, IVPP V 26561, IVPP V 26560, IVPP V 26257, IVPP V 26258, IVPP V 26259.1, IVPP V 26259.2 and IVPP V 26259. The specimen MCUGB 05-01-09 is stored in Museum of China University of Geosciences (Beijing), China.
